# MHD Boundary Layer Slip Flow and Heat Transfer of Ferrofluid along a Stretching Cylinder with Prescribed Heat Flux

**DOI:** 10.1371/journal.pone.0083930

**Published:** 2014-01-22

**Authors:** Muhammad Qasim, Zafar Hayat Khan, Waqar Ahmad Khan, Inayat Ali Shah

**Affiliations:** 1 Department of Mathematics, COMSATS Institute of Information Technology, Park Road, Chak Shahzad, Islamabad, Pakistan; 2 School of Mathematical Sciences, Peking University, Beijing, People's Republic of China; 3 Department of Engineering Sciences, PN Engineering College, National University of Sciences and Technology, Karachi, Pakistan; 4 Department of Mathematics, Islamia College Peshawar (CU), Khyber Pakhtunkhwa, Pakistan; University of Adelaide, Australia

## Abstract

This study investigates the magnetohydrodynamic (MHD) flow of ferrofluid along a stretching cylinder. The velocity slip and prescribed surface heat flux boundary conditions are employed on the cylinder surface. Water as conventional base fluid containing nanoparticles of magnetite (Fe_3_O_4_) is used. Comparison between magnetic (Fe_3_O_4_) and non-magnetic (Al_2_O_3_) nanoparticles is also made. The governing non-linear partial differential equations are reduced to non-linear ordinary differential equations and then solved numerically using shooting method. Present results are compared with the available data in the limiting cases. The present results are found to be in an excellent agreement. It is observed that with an increase in the magnetic field strength, the percent difference in the heat transfer rate of magnetic nanoparticles with Al_2_O_3_ decreases. Surface shear stress and the heat transfer rate at the surface increase as the curvature parameter increases, i.e curvature helps to enhance the heat transfer.

## Introduction

Owing to numerous industrial and engineering applications, the MHD flow analysis of the nanofluids (mixture of fluids and nanoparticles) has been increased in recent years. Many ordinary fluids like water, ethylene glycol, and mineral oils have poor thermal characteristics in comparison with metals, non-metals and their oxides. Choi [1] was the first who experimentally verified that addition of nanoparticles in conventional base fluids appreciably enhanced the thermal conductivity. Heat transfer has enormous applications in many manufacturing processes for example, in microelectronics, fuel cells, hybrid-powered engines nuclear reactors, transportations, biomedicine/pharmaceutical processes and pasteurization of food. In these processes, heat transfer takes place through some heat transfer devices; such as heat exchangers, evaporators, condensers and heat sinks. Increasing the heat transfer efficiency of these devices is desirable to minimize the space. Further in most of the heat transfer systems the working fluid is circulated by a pump, so the associated power consumption should be minimized [2]. A variety of nuclear reactor designs featured by enhanced safety and improved economics are being proposed by the nuclear power industry around the world to more realistically solve the future energy supply shortfall. In order to secure safety and economics, nanofluid coolants exhibiting improve thermal performance are being considered as a new key technology [3]. Thermal conductivity of nanofluids depends on many factors such as particle volume fraction, particle material, particle size and shape, base fluid material and temperature [2].

Boundary layer flows over stretching surfaces has promising applications in polymer processing, continuous casting, drawing of plastic sheets, stretching of plastic films and in the condensation process of metallic plates in cooling bath etc. In all these processes the quality of final product strongly depends upon rate of cooling. An electrically conducting fluid subject to magnetic field is useful in controlling the rate of cooling. The cooling rate is controlled by drawing continuous strips and filaments in an electrically conducting nanofluid [4] Such type of electrically conducting nanofluids in which nanoparticles (Magnetite, Hematite, Cobalt Ferrite, or some other compounds containing iron) are suspended in the conventional base fluids, are termed as ferrofluids. Recently, various contributions dealing with the flow of nanofluids over stretching surfaces have been reported [5]–[13]. The study of boundary layer flow along a stretching cylinder has been conducted by Ishak and Nazar [14]. The characteristics of heat transfer on a stretching cylinder with prescribed heat flux have been examined by Bachok and Ishak [15]. Finite difference scheme namely Keller box method has been utilized for the numerical solutions of the resulting differential system. Slip effects on the chemical reactive solute transfer in boundary layer flow along a stretching cylinder has been examined by Mukhopadhyay [16]. In another paper, Mukhopadhyay [17] analyzed the magnetohydrodynamic (MHD) boundary layer slip flow along a stretching cylinder. Ashornejad et al. [18] discussed the nanofluid flow and heat transfer due to a stretching cylinder in the presence of magnetic field. They solved the resulting differential system numerically by shooting technique along with fourth order Runge-Kutta integration scheme.

To the best of our knowledge, no one has investigated the boundary layer flow of a ferrofluid. along a stretching cylinder with slip velocity. Here we are interested in examining the magnetohydrodynamic (MHD) flow of ferrofluid along a stretching cylinder in presence of velocity slip condition and prescribed surface heat flux. Water as a conventional base fluid containing nanoparticles of magnetite (Fe_3_O_4_) has been considered. Further a comparison between magnetic (Fe_3_O_4_) and non-magnetic (Al_2_O_3_) nanoparticles is also made.

### Mathematical Formulation

**Figure 1:**
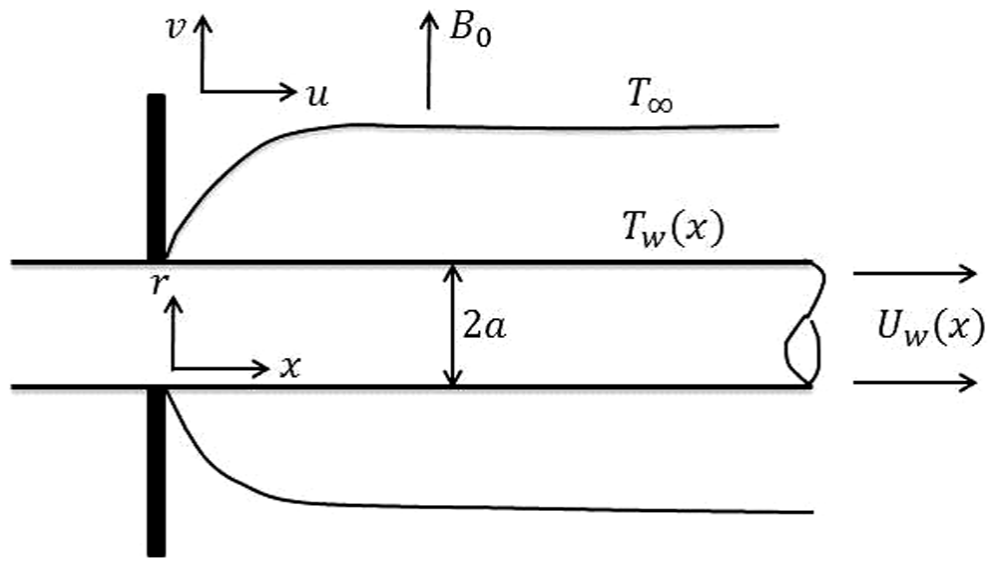
Physical model and coordinate system.

We consider the axisymmetric boundary layer flow over a circular cylinder of radius *a* placed in a ferrofluid. We assumed that uniform magnetic field of intensity 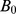 acts in the radial direction and under the assumption of small magnetic Reynolds number the effect of induced magnetic field is negligible. Further, it is assumed that cylinder is being stretched in the axial direction with velocity 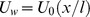 and the surface of the cylinder is subjected to a prescribed heat flux 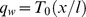, where 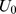 and 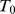 are constants, and 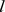 is the characteristics length (See Fig. 1). Under these assumptions the boundary layer equations governing the flow and heat transfer (in absence of viscous dissipation) are [15]–[19]

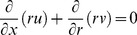
(1)

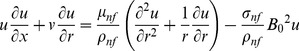
(2)

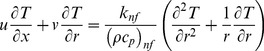
(3)


The boundary conditions are
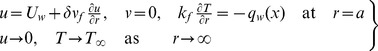
(4)


Here 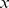 and 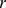 are coordinates measured in the radial and axial direction of the cylinder, respectively, 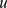 and 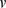 are the velocity components along 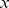 and 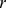 directions. Temperature is denoted by 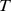, 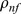 is the density of the nanofluid, 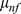
is the dynamic viscosity of the nanofluid and 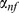 is the thermal diffusivity of the nanofluid given by
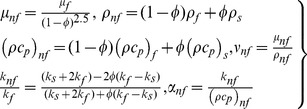
(5)


In which, 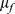 is the viscosity of the fluid fraction, 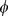 is the solid volume fraction of the nano fluid, 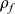 is the reference density of the fluid fraction, 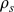 is the reference density of the solid fraction, 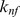 is the thermal conductivity of the nanofluid, 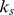 is the thermal conductivity of the solid fraction, 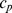 is the specific heat at constant pressure.

For simplicity, following variables are defined
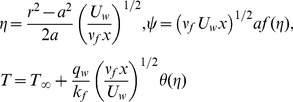
(6)where 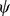 is the stream function defined as 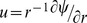 and 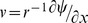 which identically satisfies the continuity eq (1). Substituting (6) in (2)–(5) we have
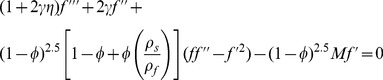
(7)

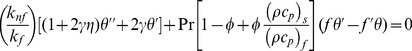
(8)

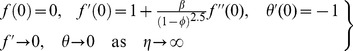
(9)where 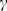 is the curvature parameter, Pr is the Prandtl number, *M* is the magnetic term and 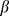 is the dimensionless slip parameter, respectively defined by
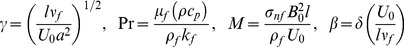
(10)


It is important to note that for a cylinder 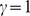 and for a plate 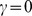. Physical quantities of interest are the skin friction coefficient 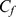 and the local Nusselt number 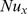. These can be written as
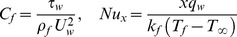
(11)in which 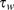 is the skin friction and 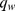 is the heat flux from the plate which are given by
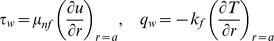



Substituting Eq. (6) into Eq. (12), we have
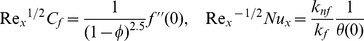
(12)


Note that for the pure fluid 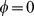, in absence of slip condition 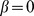 and magnetic field 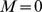 Eqs. (7)–(10) reduces to [15].

### Numerical procedure

The self-similar non-linear differential equations (7) and (8) subjected to the boundary conditions (9) are solved using the shooting technique, by converting the boundary value problem (BVP) into initial value problem (IVP). The shoot values are selected in such way that the far field boundary conditions, i.e., at 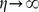 is satisfied at a finite value, say 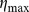. Introducing the new set of dependent variables, *p*, *q*, and *z*, we set the following first-order system
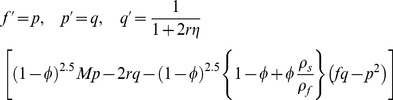
(13)and
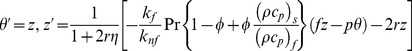
(14)with the boundary conditions
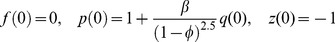
(15)


Now to solve the initial value problem (13) and (14), we need values for 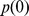, i.e., 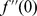 and 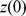, i.e., 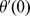 but no such values are known in advance. The initial guesses values of 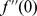 and 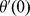 are chosen and fourth order Runge-Kutta method is applied to obtain a solution. We compared the calculated values of 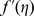 and 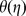 at the far field boundary condition 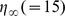 with the given boundary condition 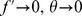 and the values of 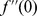 and 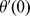 are adjusted using Secant method for better approximation. The step-size is taken as 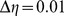 and accuracy to the fifth decimal place as the criterion of convergence.

To validate the accuracy of the proposed numerical scheme, a comparison of the obtained results corresponding to the Nusselt number is made with the available literature [15] in Table 1 and is found to in good agreement.

**Table 1:** Comparison of the present results with the literature for the Nusselt number with 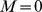 and 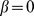.

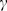	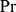	Bachok (2010) [15]	Present results
0.0	0.72	1.2367	1.23664
	1.0	1.0000	1.00000
	6.7	0.3333	0.33330
	10	0.2688	0.26876
1.0	0.72	0.8701	0.87018
	1.0	0.7439	0.74406
	6.7	0.2966	0.29661
	10.0	0.2422	0.24217

## Results and Discussion

**Figure 2:**
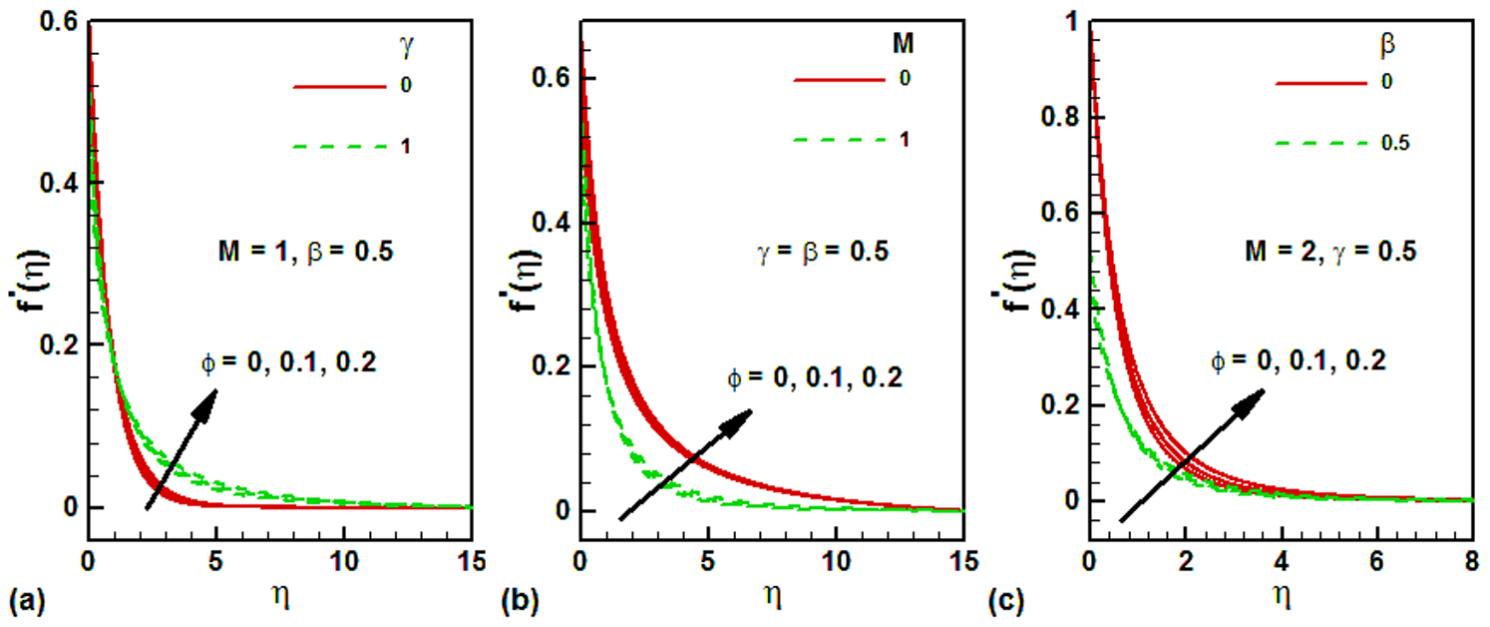
Effects of several parameters on the dimensionless velocity along vertical wall for different values of magnetite nanoparticle volume fraction.

The thermophysical properties of the base fluids water and the nanoparticle magnetite are listed in Table 2. The variation of dimensionless velocity with several parameters for different values of nanoparticle volume fraction is shown in Figs. 2(a)–2(c) for water-based magnetite ferrofluids. For a flat plate 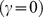, the dimensionless velocity is smaller than a vertical cylinder 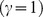 within the velocity boundary layer, as shown in Fig. 2(a). It is important to note that the dimensionless velocity increases with the nanoparticle volume friction in each case. Figure 2(b) shows the effects of magnetic parameter on the dimensionless velocity along a vertical cylinder. It is found that the dimensionless velocity is higher at the surface in the absence of magnetic field and it decreases with an increase in the magnetic field. The effects of slip parameter on the dimensionless velocity for different values of magnetite nanoparticle volume fraction are depicted in Fig. 2(c) for a vertical cylinder. In the presence of magnetic field, the surface dimensionless velocity decreases with an increase in the slip parameter. The effects of magnetite nanoparticle volume fraction on the dimensionless velocity could not be observed appreciable in any case.

**Table 2:** Thermophysical properties of base fluid/water and nanoparticle/magnetite [19], [20].

Physical Properties	Water/base fluid	Magnetite/Magnetic	Al_2_O_3_/Non-magnetic
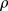 (kg/m^3^)	997	5180	3970
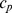 (J/kg K)	4179	670	765
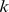 (W/m K)	0.613	9.7	40

**Figure 3:**
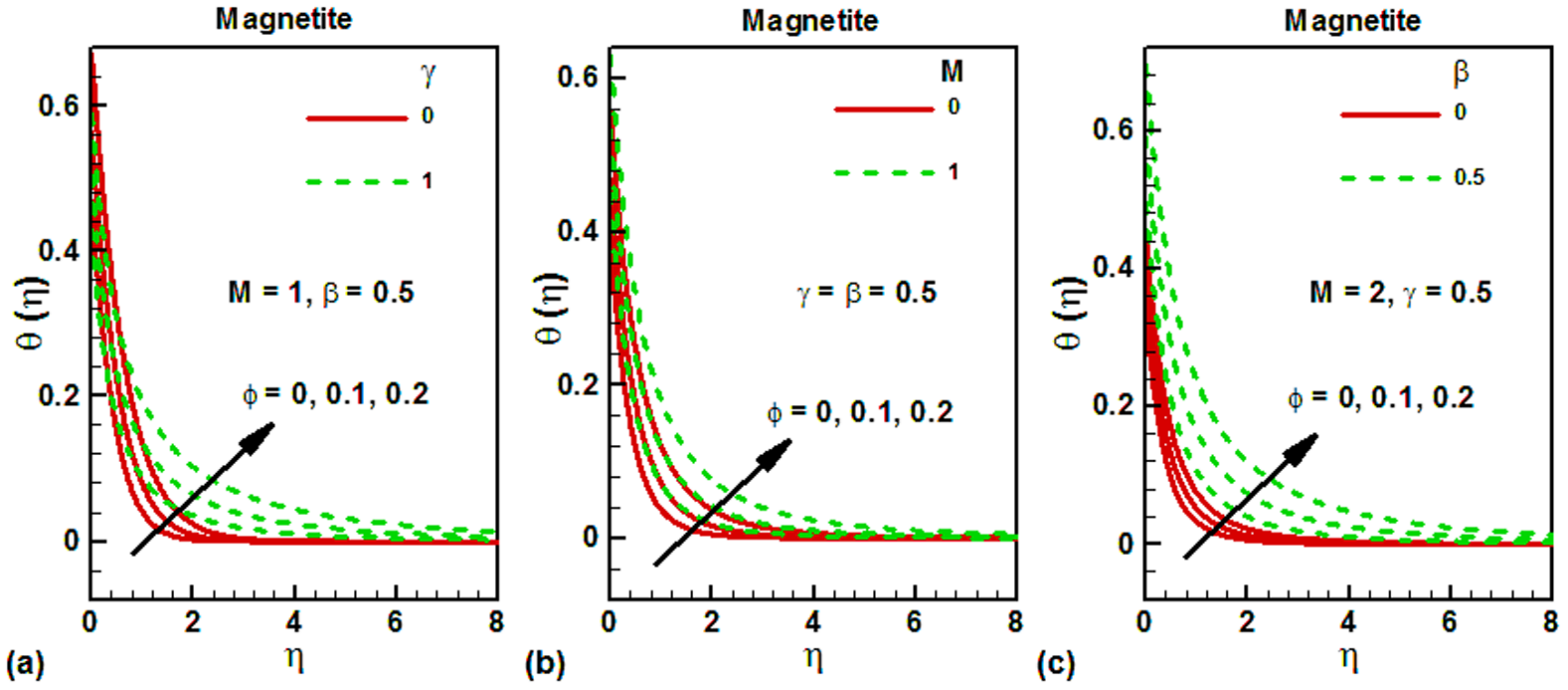
Effects of several parameters on the dimensionless temperature along vertical wall for different values of magnetite nanoparticle volume fraction.

The effects of the same parameters on the dimensionless temperature are shown in Figs. 3(a)–3(c) for water-based magnetite ferrofluids. The dimensionless temperature at the plate surface is found to be higher and converges quickly, as shown in Fig. 3(a). Due to this reason, the thermal boundary layer thickness will be smaller and the heat transfer rate from the plate surface will be higher as observed in Table 2. The thermal boundary layer thickness at the cylinder surface is larger which increases the thermal resistance to heat transfer from cylinder. The effects of magnetic field on the dimensionless temperature at the cylinder surface for various values of magnetite nanoparticle volume fraction are shown in Fig. 3(b). In the absence of magnetic field, the dimensionless temperature at the surface is smaller and increases with an increase in the magnetic field, as shown in Fig. 3(b). It is also observed that the thermal boundary layer thickness increases with magnetic field. Figure 3(c) depicts the effects of velocity slip on the dimensionless temperature of the cylinder surface for various values of magnetite nanoparticle volume fraction. It can be seen that the dimensionless temperature at the surface is lower in the absence of velocity slip and it increases with an increase in the velocity slip. This is due to decrease in the dimensionless velocity with increasing slip. The thermal boundary layer thickness is also found to increase with increasing magnetic field.

**Figure 4:**
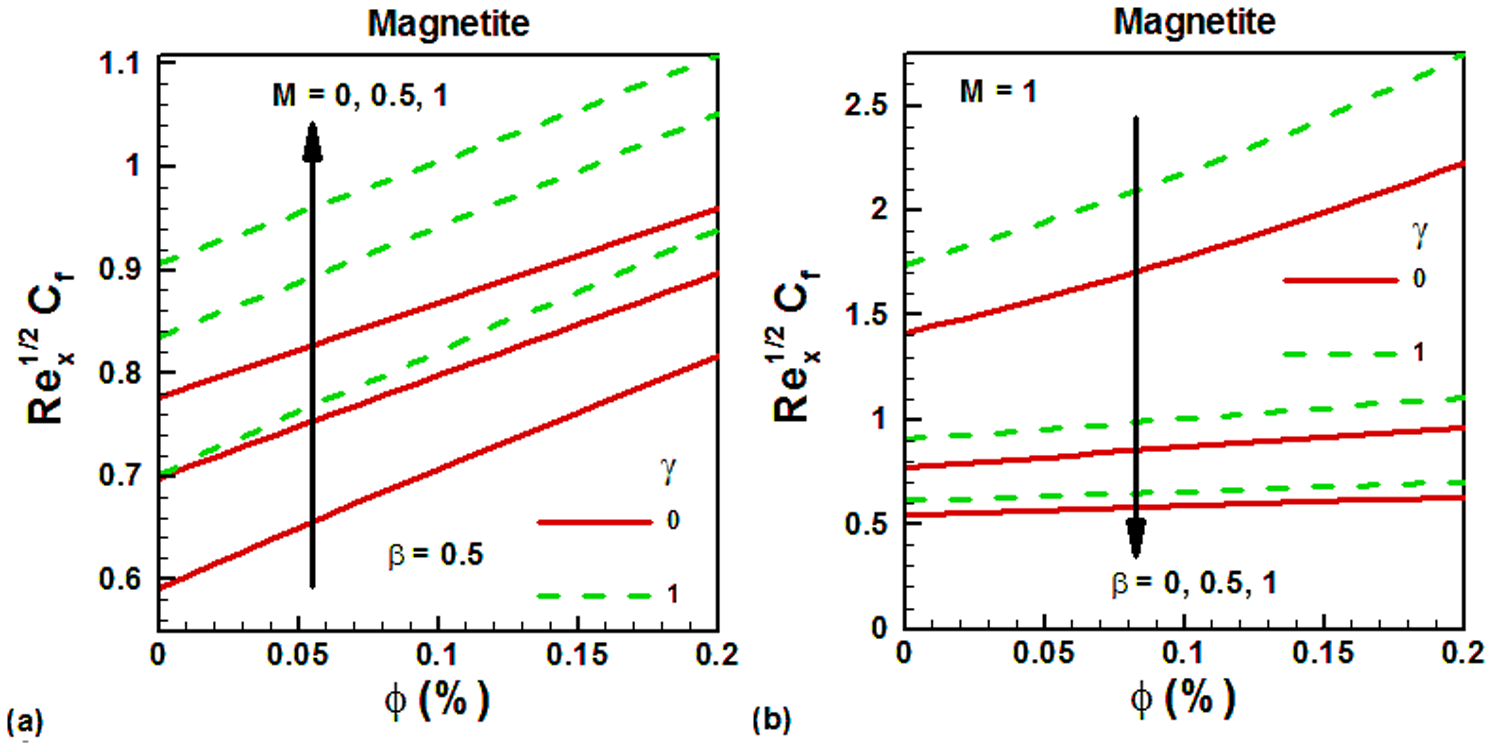
Variation of skin friction with magnetite nanoparticle volume fraction for different values of controlling parameters.

**Figure 5:**
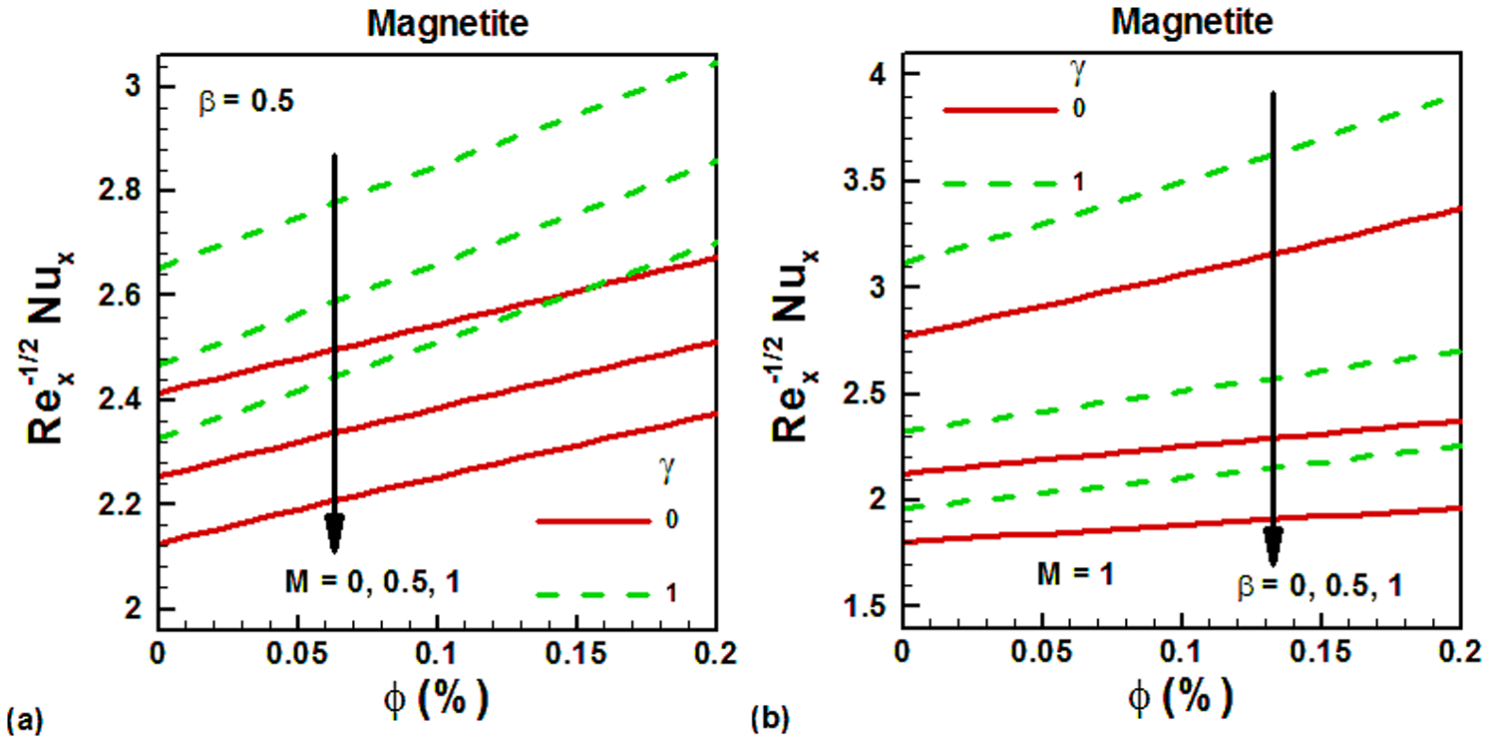
Variation of Nusselt numbers with magnetite nanoparticle volume fraction for different values of controlling parameters.

The density of nanofluids increases with increasing nanoparticle volume fraction. Due to this reason, the skin friction also increases with increasing nanoparticle volume fraction. This is shown in Figs. 4(a) and 4(b) for water-based magnetite ferrofluids. It can be seen that the skin friction is higher for the vertical cylinder in both cases due to larger surface area. In the absence of magnetic field, the skin friction is smaller and it increases with increasing magnetic field, as shown in Fig. 4(a). On the other hand, the skin friction is decreasing with slip velocity, as shown in Fig. 4(b). The variation of Nusselt numbers with magnetite nanoparticle volume fraction is shown in Figs. 5(a) and 5(b) for water-based magnetite ferrofluids. It can be seen that Nusselt numbers increase with increasing magnetite nanoparticle volume fraction in both cases. This is due to increase in the thermal conductivity of ferrofluids with increasing nanoparticle volume fraction. The effects of magnetic field on the Nusselt numbers are explored in Fig. 5(a) for both vertical plate and cylinder. It is noticed that the Nusselt numbers are higher for vertical cylinder. In the absence of magnetic field, the Nusselt numbers are higher for both geometries and decrease with increasing magnetic field. The effects of velocity slip on the Nusselt numbers are shown in Fig. 5(b) for both geometries. In the absence of velocity slip, the Nusselt numbers are found to be higher and they decrease with increasing velocity slip. To validate the accuracy of the proposed numerical scheme, a comparison of the obtained results corresponding to the Nusselt number is made with the available literature [15] in Table 1 and is found to in good agreement. In Table 3, we have made comparison between magnetic and non-magnetic nanoparticles. The selected non-magnetic nanoparticle is Al_2_O_3_ whose thermal conductivity is 40 W/m-K. Due to higher thermal conductivity of Al_2_O_3_ than magnetic nanoparticles, the Nusselt numbers of Al_2_O_3_ are found to be higher in the absence of magnetic field. However, as the strength of the magnetic field is increased, the magnetic nanoparticles get aligned in one direction and exhibit higher heat transfer rates which are comparable with non-magnetic nanoparticles like Al_2_O_3_. This comparison is shown in Table 3. It can be seen that, in the absence of magnetic field, the heat transfer rates are higher for Al_2_O_3_ than other selected magnetic nanoparticles. The percent difference between Al_2_O_3_ and other magnetic nanoparticles increases with increasing solid volume fraction of nanoparticles. But when the magnetic field is applied and its strength is increasing, the magnetic nanoparticles get aligned and the percent difference with Al_2_O_3_ start decreasing.

**Table 3:** Variation of Nusselt numbers with magnetic parameter and solid volume fraction of magnetic and non-magnetic nanoparticles with 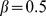 and 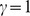.

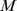	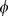	Magnetite/Magnetic	Al_2_O_3_/Non-magnetic	% difference with Al_2_O_3_
0	0	2.64809	2.64809	0
	0.05	2.74461	2.77643	0.0114
	0.1	2.84155	2.90398	0.0214
	0.2	3.03774	3.16113	0.0390
1.0	0	2.32602	2.32602	0
	0.05	2.41998	2.44199	0.0091
	0.1	2.51430	2.55845	0.0172
	0.2	2.70789	2.79984	0.0328
2	0	2.11328	2.11328	0
	0.05	2.20143	2.21883	0.0078
	0.1	2.29118	2.32663	0.0152
	0.2	2.47968	2.55661	0.0300

### Conclusions

The present study investigates the magnetohydrodynamics flow and heat transfer of ferrofluid along a stretching cylinder with slip velocity. The main observations of this study are:

- Dimensionless velocity decreases with an increase in the slip parameter.
- Surface shear stress and the heat transfer rate at the surface increase as the curvature parameter increases. Hence curvature helps to enhance the heat transfer.
- Surface shear stress also increases with increasing nanoparticle volume fraction.
- The thermal boundary layer thickness increases with magnetic field.
- Nusselt number decreases by increasing velocity slip.
- Nusselt numbers increase with increasing magnetite nanoparticle volume fraction.
- With an increase in the magnetic field strength, the percent difference in the heat transfer rate of magnetic nanoparticles with Al_2_O_3_ decreases.
