# Correction: MHD Boundary Layer Slip Flow and Heat Transfer of Ferrofluid along a Stretching Cylinder with Prescribed Heat Flux

**DOI:** 10.1371/journal.pone.0100462

**Published:** 2014-06-11

**Authors:** The PLOS ONE Staff

Equations 4, 9, and 15, and Table 3 are incorrect. The authors have provided the correct versions here.
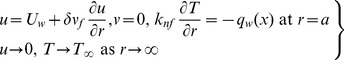
(4)

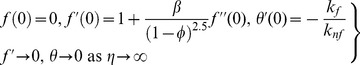
(9)

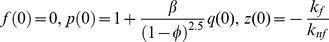
(15)


**Table 3:** Variation of Nusselt numbers with magnetic parameter and solid volume fraction of magnetic and non-magnetic nanoparticles with 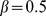 and 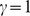.

M	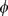	Magnetite/Magnetic	Al_2_O_3_/Non-magnetic	% difference with Al_2_O_3_
0	0	2.64809	2.64809	0
	0.05	3.10186	3.19428	0.02893
	0.1	3.61485	3.82424	0.05475
	0.2	4.85606	5.40124	0.10093
1	0	2.32602	2.32602	0
	0.05	2.73498	2.80950	0.02652
	0.1	3.19854	3.49159	0.08393
	0.2	4.32876	4.78391	0.09514
2	0	2.11328	2.11328	0
	0.05	2.48799	2.55276	0.02537
	0.1	2.91470	3.06393	0.04870
	0.2	3.96396	4.36833	0.09256
